# From “scientific leadership” to sustainable innovation: the shaping mechanism of talent ecosystem and green innovation capability in Chinese knowledge-intensive firms

**DOI:** 10.3389/fpsyg.2026.1811379

**Published:** 2026-05-15

**Authors:** Rongcheng Liang

**Affiliations:** 1 Emergency Management Training Department, Party School of Shandong Provincial Committee of the Communist Party of China (Shandong Academy of Governance), Jinan, China; 2 School of International Relations and Public Affairs, Fudan University, Shanghai, China

**Keywords:** green innovation capability, knowledge-intensive firms, organizational learning, scientific leadership, talent ecosystem

## Abstract

In the context of global sustainable development, knowledge-intensive firms face unprecedented challenges in cultivating green innovation capabilities. Drawing upon the theoretical foundations of talent ecosystem theory and dynamic capability theory, this study investigates the shaping mechanism through which talent ecosystems influence green innovation capability in Chinese knowledge-intensive enterprises. By analyzing the interplay among scientific leadership, talent density, and organizational learning climate, it develops a conceptual framework that elucidates how human capital serves as the core driving force for organizational sustainable development. Our findings reveal that a robust talent ecosystem, characterized by mission-driven leadership and collaborative knowledge networks, significantly enhances firms’ green innovation performance through the mediation of organizational learning mechanisms. Furthermore, scientific leadership emerges as a critical boundary condition that amplifies the positive relationship between talent ecosystem characteristics and green innovation outcomes. This research contributes to the social sustainability literature by highlighting the critical role of talent development in achieving environmental sustainability goals, offering practical implications for managers seeking to build sustainable competitive advantages through strategic human resource management. The study advances our understanding of the micro-foundations of sustainable innovation and provides actionable insights for organizations navigating the transition toward environmentally responsible business models.

## 1 Introduction

The global imperative for sustainable development has fundamentally transformed the competitive landscape for contemporary enterprises (Adams et al., 2016). As environmental concerns intensify and stakeholder expectations evolve, organizations are increasingly compelled to integrate sustainability principles into their core strategic frameworks. Among the various dimensions of sustainable development, green innovation has emerged as a critical capability that enables firms to reconcile environmental responsibility with economic performance, thereby achieving the dual objectives of ecological preservation and competitive advantage (Huang and Li, 2017). The transition toward sustainable business models requires organizations to fundamentally rethink their approach to innovation, moving beyond incremental improvements toward transformative changes that address systemic environmental challenges.

Knowledge-intensive firms, characterized by their reliance on intellectual capital and specialized expertise, occupy a particularly significant position in the transition toward sustainable business models. These organizations, encompassing sectors such as information technology, biotechnology, advanced manufacturing, and professional services, possess inherent advantages in developing green innovations due to their cognitive resources and problem-solving capabilities. The knowledge-based nature of these firms means that their competitive advantages derive primarily from the capabilities of their workforce rather than from physical assets or market position (Barney, 1991). However, the realization of this potential depends critically on the effective cultivation and deployment of human capital within an organizational context that fosters creativity, collaboration, and environmental consciousness.

The concept of talent ecosystem, rooted in the seminal work of organizational ecologists and strategic human resource management scholars. This perspective, grounded in the resource-based view of the firm and organizational ecology theory, has gained increasing attention in international management research, provides a compelling lens for understanding how firms can systematically develop the human capabilities necessary for sustainable innovation. Unlike traditional human resource management approaches that focus primarily on individual employee attributes and job-person fit, the talent ecosystem perspective emphasizes the interconnected nature of human capital within organizations, recognizing that innovation outcomes emerge from complex interactions among diverse actors, structures, and processes (Liang, 2024). This perspective draws upon ecological metaphors to conceptualize organizational talent as comprising interconnected populations of individuals whose collective interactions generate emergent capabilities that transcend the sum of individual contributions.

This study is particularly motivated by the theoretical contributions of Professor Jianfeng Peng, a pioneering scholar in strategic human resource management and founder of China Stone Management Consulting Group, and his research on the formation mechanisms of scientific leadership in organizations. Professor Peng’s extensive work on talent ecosystem theory, developed through decades of research and consulting practice with leading Chinese enterprises including Huawei, TCL, and New Hope Group, provides a robust theoretical foundation for understanding how organizations can cultivate the human capabilities necessary for sustainable competitive advantage. Drawing upon his conceptualization of talent ecosystem dynamics and the doctoral research of Liang (2024) on the emergence of scientific leadership, this paper develops an integrative framework that connects talent ecosystem characteristics with green innovation capabilities in the context of Chinese knowledge-intensive firms. While this study draws upon Chinese theoretical developments and empirical context, the proposed framework is grounded in internationally-established theories including the resource-based view, dynamic capabilities theory, and organizational learning theory, ensuring broader relevance to organizational psychology and innovation management research. This framework directly engages with contemporary international debates on the micro-foundations of dynamic capabilities, the role of human capital in fostering workplace creativity, and the leadership mechanisms that enable sustainable innovation in knowledge-intensive environments. By integrating talent ecosystem theory with organizational psychology and innovation management literatures, this study responds to recent calls for cross-level research that connects individual creativity, team processes, and organizational sustainability outcomes. At the same time, by explicitly integrating recent advances in workplace creativity research, this framework positions talent ecosystem dynamics as antecedents of creative self-efficacy and psychological safety—two psychological mechanisms consistently linked to innovative work behavior (Zhao and Ren, 2025; Qian et al., 2025).

The Chinese context offers unique insights into the relationship between talent management and sustainable innovation. China’s rapid economic development, coupled with increasing environmental pressures and policy initiatives promoting green transformation, has created a distinctive institutional environment where knowledge-intensive firms must navigate complex challenges in building innovation capabilities. The Chinese government’s emphasis on ecological civilization construction and the dual carbon goals of peaking carbon emissions before 2030 and achieving carbon neutrality before 2060 has created both regulatory pressures and market opportunities for green innovation. Furthermore, the cultural emphasis on collective achievement and hierarchical relationships influences how talent ecosystem function and evolve within Chinese organizations.

The primary objective of this research is to elucidate the mechanisms through which talent ecosystems shape green innovation capabilities in knowledge-intensive firms. Specifically, it seeks to address three interrelated research questions: (1) What are the key dimensions of talent ecosystems that influence green innovation capability? (2) How does scientific leadership function as a catalyst in translating talent ecosystem advantages into innovation outcomes? (3) What organizational mechanisms mediate the relationship between talent ecosystem characteristics and green innovation performance? By addressing these questions, this study makes several contributions to the literature. Specifically, this research contributes to current debates on workplace creativity by examining how talent ecosystem characteristics foster creative synthesis; advances understanding of future-ready teams by identifying the dynamic capabilities that enable adaptive innovation; and illuminates the psychological and team-level mechanisms—including organizational learning and psychological safety—that support innovative work behavior. First, it extends the application of talent ecosystem theory to the domain of environmental sustainability, demonstrating how human capital development can serve as a foundation for green innovation. Second, it integrates insights from leadership studies and innovation management to develop a more nuanced understanding of the micro-foundations of sustainable competitive advantage. Third, it provides empirical evidence from the Chinese context, contributing to the growing body of research on sustainability management in emerging economies.

## 2 Literature review

### 2.1 Talent ecosystem theory and organizational sustainability

The talent ecosystem concept has evolved significantly since its introduction into the management literature. Drawing upon ecological metaphors, scholars have conceptualized organizational talent as comprising interconnected populations of individuals whose collective interactions generate emergent capabilities that transcend the sum of individual contributions. This perspective emphasizes the importance of diversity, interdependence, and co-evolution in understanding how organizations develop and sustain competitive advantages through human capital. The ecosystem metaphor is particularly apt because it captures the dynamic, evolving nature of organizational talent and the complex interactions that produce innovation outcomes.

The talent ecosystem is an open, dynamic, and networked talent management model. It regards the organization as an organic ecological environment, where talents can freely flow within and outside the organization, emphasizing the establishment of symbiotic relationships with external partners, universities, research institutions, etc. Its core features are blurred boundaries and resource sharing, emphasizing the construction of a long-term talent supply chain and the collaborative development of ecological partners. The talent ecosystem is outward oriented and ecological, focusing on the interaction between organizations and the external environment, as well as the construction of talent networks. Unlike human capital climate, which focuses on employees’ shared perceptions of HR practices and organizational support, the talent ecosystem perspective emphasizes the dynamic, networked nature of talent interactions and co-evolutionary processes that generate emergent capabilities. The high-performance work system is introverted and systematic, focusing on the integration and optimization of internal human resource practices. The former adapts to uncertainty and innovation needs, while the latter pursues stability and efficiency improvement (Delery and Roumpi, 2017; Kramar, 2014).

Recent scholarship has identified several critical dimensions of talent ecosystems that influence organizational outcomes. Talent density refers to the concentration of high-capability individuals within specific organizational units or networks, creating knowledge spillovers and elevating collective performance standards. High talent density creates a competitive environment where individuals are motivated to perform at their best while also providing opportunities for learning through observation and collaboration. The psychological mechanisms underlying this effect include social comparison processes that motivate performance improvement and observational learning that facilitates skill acquisition. Talent flow captures the dynamic nature of human capital movement, including internal rotation, external recruitment, and knowledge transfer processes that continuously reshape the ecosystem’s composition and capabilities. Active talent flow prevents organizational rigidity and ensures that the ecosystem remains responsive to changing environmental demands. Talent structure encompasses the configuration of skills, experiences, and cognitive styles within the ecosystem, influencing the range of problems that can be effectively addressed and the types of innovations that can be generated.

The relationship between talent ecosystems and organizational sustainability has received increasing attention in recent years. Scholars have argued that sustainable competitive advantage requires not merely the accumulation of talented individuals but the cultivation of an ecosystem that enables continuous learning, adaptation, and innovation (Alshukri et al., 2024). This perspective aligns with the resource-based view’s emphasis on resource bundles and organizational capabilities while providing more specific insights into the human capital mechanisms underlying sustainable performance (Barney, 1991). From a social sustainability perspective, the talent ecosystem approach highlights the importance of developing human capital as a foundation for long-term organizational success, recognizing that investments in people create value not only for the organization but for society as a whole. These insights resonate with emerging international research that examines how talent configurations influence workplace creativity and innovative work behavior across diverse institutional contexts. The talent ecosystem perspective offers a particularly valuable lens for understanding creativity as an emergent property of interconnected talent networks, moving beyond individual-level trait approaches to examine how structural features of talent systems----density, flow, and diversity----collectively shape the creative capacity of organizations. This emergent view aligns with recent evidence that complementary fit and psychological safety energize employee creativity by reducing cognitive load and fostering resource investment (Zhao and Ren, 2025), while learning-oriented cultures enhance the creative self-efficacy that translates individual capability into collective innovation (Qian et al., 2025).

### 2.2 Green innovation capability in knowledge-intensive firms

Green innovation, defined as the development and implementation of new products, processes, services, or management methods that reduce environmental impact and enhance ecological sustainability, has become a central concern for both academic researchers and practitioners (Rennings, 2000). Unlike traditional innovation focused primarily on economic returns, green innovation must balance environmental effectiveness with commercial viability, creating unique challenges for organizations seeking to develop this capability. The multi-dimensional nature of green innovation requires organizations to consider not only technical feasibility but also environmental impact, regulatory compliance, market acceptance, and long-term sustainability.

Knowledge-intensive firms possess distinctive characteristics that influence their green innovation trajectories. Their reliance on specialized expertise and cognitive capabilities enables them to identify environmental problems and develop technically sophisticated solutions. These firms are typically characterized by high levels of R&D investment, educated workforces, and knowledge-based competitive advantages that position them well for developing innovative solutions to environmental challenges. However, the translation of technical potential into innovation outcomes depends on organizational factors such as strategic orientation, resource allocation priorities, and cultural values related to environmental responsibility.

Research has identified multiple dimensions of green innovation capability. Green product innovation involves the development of environmentally friendly products that reduce resource consumption, minimize pollution, or enable recycling and reuse. This dimension requires firms to rethink product design, material selection, and manufacturing processes from an environmental perspective. Green process innovation focuses on improving production and operational methods to reduce environmental impact while maintaining or enhancing efficiency. Process innovations may involve adopting cleaner technologies, optimizing resource utilization, or implementing closed-loop systems that minimize waste. Green management innovation encompasses organizational and managerial innovations that embed sustainability principles into core business practices and decision-making processes. This dimension recognizes that technical innovations must be supported by organizational systems and cultures that prioritize environmental sustainability. These multi-dimensional conceptualizations of green innovation have been validated across diverse national contexts, with comparative studies highlighting how institutional environments shape the relative emphasis on product, process, and management innovations. The international literature further underscores that knowledge-intensive firms face common challenges in aligning green innovation strategies with dynamic talent management practices, regardless of regional institutional differences.

### 2.3 Scientific leadership and talent development

The concept of scientific leadership, as elaborated in the research of Liang (2024), refers to a distinctive form of organizational leadership characterized by deep technical expertise, systems thinking capability, and the ability to mobilize collective intelligence toward complex problem-solving. Unlike traditional administrative leadership focused primarily on resource allocation and coordination, scientific leadership emphasizes knowledge creation, intellectual stimulation, and the cultivation of next-generation talent. Scientific leaders are distinguished by their ability to understand complex technical problems, envision innovative solutions, and inspire others to pursue ambitious goals.

Scientific leadership emphasizes a leadership style based on evidence-based decision-making, data-driven, and systematic methods. It focuses on making decisions through empirical research, data analysis, and scientific methods, reducing subjective judgments and intuitive dependence, and pursuing objectivity and repeatability in management. This distinguishes scientific leadership from transformational leadership, which focuses primarily on vision articulation and inspirational motivation to unleash follower potential, and from expert leadership, which centers narrowly on applying specialized technical knowledge to solve domain-specific problems. While transformational leaders ask “how to inspire commitment” and expert leaders ask “how to solve technical problems”, scientific leaders ask “how to make correct decisions through systematic inquiry”. Scientific leaders are more like ‘researchers’, using hypothesis testing and evidence evaluation to guide organizational behavior. Scientific leadership revolves around methodology and evidence, transformative leadership revolves around vision and motivation, and expert leadership revolves around professional knowledge. Scientific leadership focuses on “how to make correct decisions”, transformative leadership focuses on “how to unleash potential”, and expert leadership focuses on “how to solve technical problems”.

Scientific leaders serve multiple critical functions within talent ecosystems. They act as knowledge brokers, connecting diverse expertise domains and facilitating the integration of specialized insights into coherent innovation strategies. In complex innovation environments, where solutions often require combining knowledge from multiple disciplines, the ability to bridge different expertise domains becomes essential. Scientific leaders function as role models, demonstrating through their own behavior the values of intellectual curiosity, rigorous inquiry, and continuous learning that characterize high-performance knowledge work (Güttel et al., 2023). Their personal example sets the tone for the entire organization and establishes expectations for professional excellence. Perhaps most importantly, they serve as talent cultivators, actively developing the capabilities of emerging leaders and creating the conditions for scientific leadership to proliferate throughout the organization. In doing so, scientific leaders directly foster workplace creativity by establishing psychological safety and intellectual stimulation—two conditions consistently identified in international research as essential for creative expression and innovative work behavior. Their emphasis on evidence-based inquiry and systematic problem-solving creates an environment where employees feel encouraged to experiment, take intellectual risks, and engage in the collaborative knowledge-sharing processes that underpin collective creativity. These leadership functions directly echo the team-level mechanisms identified in recent research, where knowledge sharing and creative self-efficacy sequentially channel leadership influence into innovative work behavior (Ni et al., 2025).

The relationship between scientific leadership and green innovation is particularly salient in knowledge-intensive contexts. Environmental challenges typically require interdisciplinary expertise spanning multiple technical domains, making the integrative function of scientific leadership especially valuable. Green innovation often involves addressing complex problems at the intersection of materials science, engineering, environmental science, and business strategy, requiring leaders who can understand and synthesize insights from diverse fields. Furthermore, the long-term orientation and systems perspective characteristic of scientific leaders align well with the temporal and complexity requirements of sustainable innovation. Unlike short-term profit maximization, green innovation requires sustained commitment to environmental goals and the ability to navigate trade-offs between immediate costs and long-term benefits (Bansal, 2005).

## 3 Theoretical framework and hypotheses

**Figure 1:**
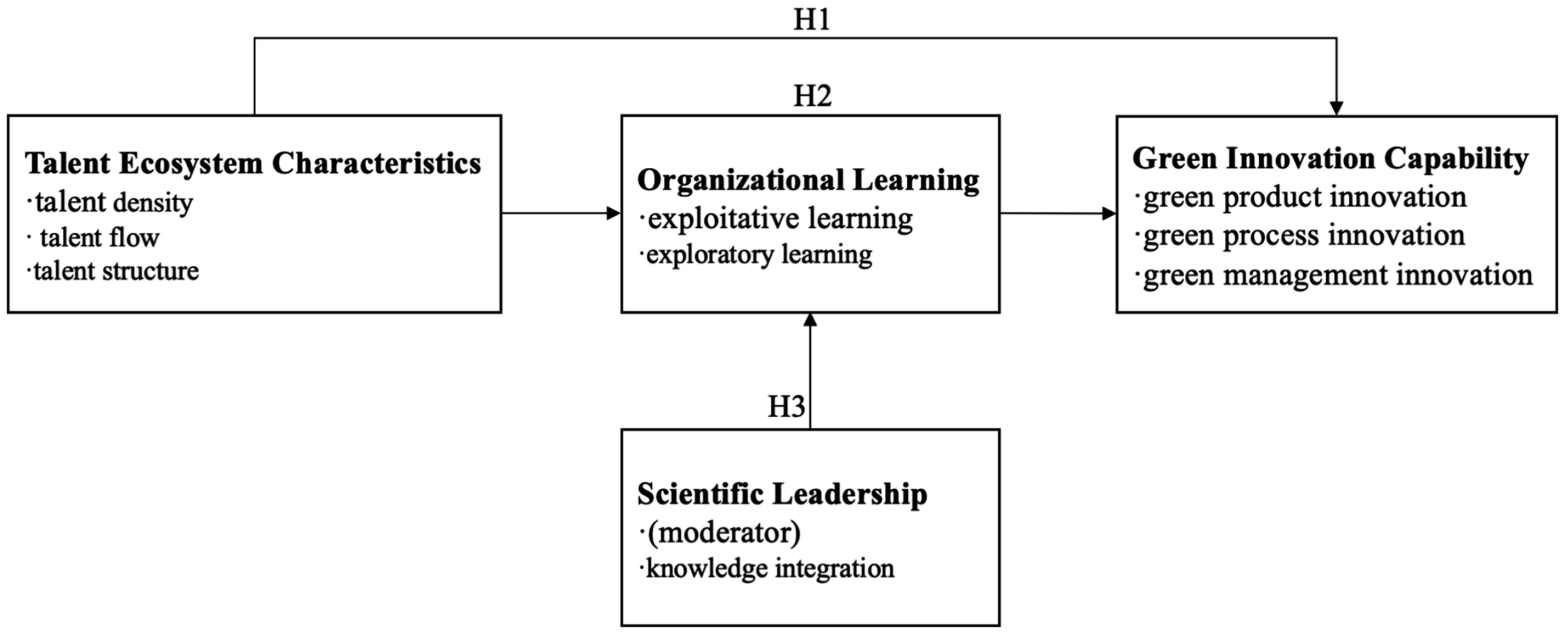
Conceptual framework of the relationship between talent ecosystem and green innovation capability.

Building upon the theoretical foundations reviewed above, this study proposes an integrative framework that elucidates the mechanisms through which talent ecosystems influence green innovation capability in knowledge-intensive firms. The framework, illustrated in Figure 1, posits that talent ecosystem characteristics (talent density, talent flow, and talent structure) influence green innovation capability through the mediation of organizational learning mechanisms, with scientific leadership serving as a critical moderating factor that amplifies these relationships. This integrative approach allows us to examine not only the direct effects of talent ecosystem characteristics but also the processes through which these effects are realized and the conditions under which they are most pronounced.

### 3.1 Talent ecosystem characteristics and green innovation capability

Talent density within knowledge-intensive firms creates the cognitive foundation for green innovation. High concentrations of talented individuals generate knowledge spillovers, elevate performance standards, and create competitive dynamics that stimulate creative problem-solving. In the context of environmental sustainability, talent density enables organizations to assemble the diverse expertise required to address complex ecological challenges, from materials science and engineering to environmental policy and stakeholder management. When talented individuals work in close proximity, whether physical or virtual, they create opportunities for knowledge exchange, collaborative problem-solving, and creative synthesis that can lead to breakthrough innovations. From a cognitive perspective, high talent density expands the organization’s collective cognitive repertoire, enabling more diverse problem representations and solution pathways.

Talent flow contributes to green innovation capability by continuously refreshing the organization’s knowledge base and bringing external perspectives into the innovation process. External recruitment introduces new ideas and approaches developed in different organizational contexts, while internal mobility facilitates knowledge transfer across functional boundaries and promotes the cross-pollination of insights relevant to sustainable innovation (Liang, 2024). The dynamic nature of talent flow prevents organizational rigidity and maintains the ecosystem’s adaptive capacity in response to evolving environmental requirements. Organizations with active talent flow are better positioned to incorporate emerging knowledge about green technologies, environmental regulations, and sustainability best practices into their innovation activities.

Talent structure influences green innovation through its effects on the range of problems that can be addressed and the types of solutions that can be generated. Diverse talent structures, encompassing varied disciplinary backgrounds, functional experiences, and cognitive styles, enhance the organization’s ability to identify environmental opportunities and develop innovative responses. Complementary expertise configurations enable the integration of technical, market, and regulatory knowledge necessary for successful green innovation. A well-structured talent ecosystem includes not only technical experts but also individuals with market knowledge, regulatory understanding, and stakeholder management capabilities, creating the multidisciplinary teams necessary for addressing complex sustainability challenges.

Based on these considerations, it proposes:

*H1*: Talent ecosystem characteristics (density, flow, and structure) are positively related to green innovation capability in knowledge-intensive firms.

### 3.2 The mediating role of organizational learning

Organizational learning serves as a critical mechanism through which talent ecosystem characteristics translate into green innovation capabilities. The presence of talented individuals creates opportunities for learning, but the realization of these opportunities depends on organizational processes that facilitate knowledge sharing, integration, and application. Organizational learning encompasses the processes through which organizations acquire, interpret, and apply knowledge to improve their capabilities and performance. In the context of green innovation, organizational learning involves developing new knowledge about environmental challenges, green technologies, and sustainable business models, as well as integrating this knowledge into innovation activities.

Exploitative learning, focused on refining and extending existing knowledge, enables organizations to build upon their accumulated expertise in developing incremental green innovations. Through exploitative learning, organizations can continuously improve their environmental performance by applying proven approaches to new contexts or enhancing the efficiency of existing green technologies and processes. Exploratory learning, oriented toward discovering new possibilities and experimenting with novel approaches, supports the development of breakthrough green innovations that fundamentally transform environmental performance. The balance between exploitation and exploration, often termed organizational ambidexterity, is particularly important in the sustainability domain where firms must simultaneously optimize current operations and develop transformative solutions.

Talent ecosystem characteristics facilitate organizational learning by creating the conditions for effective knowledge exchange and collective problem-solving. High talent density generates learning opportunities through observation, imitation, and collaborative work. When talented individuals work together, they create a rich environment for knowledge sharing and skill development. These social psychological processes of knowledge exchange are fundamental to converting individual expertise into collective organizational capability. Active talent flow brings new knowledge into the organization and exposes existing members to diverse perspectives (Liang, 2024). New employees bring knowledge from their previous experiences, while internal mobility facilitates the transfer of knowledge across organizational boundaries. Diverse talent structures create the cognitive diversity necessary for creative synthesis and innovative recombination of existing knowledge. When individuals with different backgrounds and perspectives collaborate, they are more likely to generate novel ideas and solutions.

Based on this reasoning, it hypothesizes:

*H2*: Organizational learning mediates the relationship between talent ecosystem characteristics and green innovation capability.

### 3.3 The moderating role of scientific leadership

Scientific leadership amplifies the relationship between talent ecosystem characteristics and green innovation outcomes by performing several critical functions. First, scientific leaders serve as knowledge integrators, connecting diverse expertise domains and facilitating the synthesis of insights necessary for addressing complex environmental challenges. Green innovation often requires combining knowledge from multiple disciplines, and scientific leaders, with their broad technical understanding and systems thinking capabilities, are well-positioned to facilitate this integration. Second, they create psychological safety and intellectual stimulation that encourage risk-taking and experimentation essential for innovation. Innovation, particularly in the sustainability domain, involves uncertainty and the possibility of failure, and scientific leaders can create an environment where individuals feel safe to experiment and learn from setbacks. This psychological safety—the shared belief that the team is safe for interpersonal risk-taking—enables the knowledge-sharing behaviors and creative experimentation necessary for green innovation. Third, they model the values of environmental responsibility and systems thinking that orient organizational efforts toward sustainability goals.

The presence of scientific leadership moderates the talent ecosystem-green innovation relationship by enhancing the efficiency with which talent resources are converted into innovation outcomes. Organizations with strong scientific leadership are better able to identify and attract talented individuals aligned with sustainability objectives, integrate diverse expertise into coherent innovation strategies, and maintain long-term commitment to green innovation initiatives despite short-term pressures. Scientific leaders can articulate a compelling vision for sustainability that attracts talented individuals who are motivated by environmental goals. They can also create organizational structures and processes that facilitate collaboration among diverse experts and ensure that green innovation initiatives receive the resources and attention needed for success.

Based on these arguments, it proposes:

*H3*: Scientific leadership moderates the relationship between talent ecosystem characteristics and green innovation capability, such that the relationship is stronger when scientific leadership is high.

## 4 Research methodology

### 4.1 Sample and data collection

The data for this study were collected through a survey of knowledge-intensive firms in China. The sample frame included firms operating in four high-technology sectors: information technology, biotechnology, new energy, and advanced manufacturing. These sectors were selected because they represent knowledge-intensive industries where green innovation is particularly relevant and where talent management practices are likely to influence innovation outcomes. Firms were identified from industry directories and professional associations, and senior managers with knowledge of both human resource management and innovation activities were targeted as respondents. These managerial respondents provided organizational-level assessments based on their comprehensive understanding of their firms’ talent management practices and innovation activities. This study involved human subjects (enterprise managers) participating in a survey research design. Prior to data collection, informed consent was obtained from all participants, who were informed of the voluntary nature of their participation, the confidentiality of their responses, and the academic purpose of the research. The study was conducted in accordance with established ethical guidelines for social science research involving human participants. No sensitive personal information was collected, and all data were anonymized and used for academic research purposes only.

A total of 450 questionnaires were distributed, and 312 usable responses were received, representing a response rate of 69.3%. After eliminating responses with missing data or inconsistent patterns, the final sample consisted of 287 firms. The sample characteristics reflect the diversity of the knowledge-intensive sector in China, with firms ranging from small startups to large established enterprises. Among the respondents, 42% were from the information technology sector, 23% from biotechnology, 19% from new energy, and 16% from advanced manufacturing. The average firm age was 12.5 years, and the average firm size was 487 employees.

### 4.2 Measures

It should be noted that this study revised the scale based on previous research and management practices when measuring relevant constructs by using multi-item. Green innovation capability and organizational learning measures were adopted from established international scales, while talent ecosystem characteristics were adapted from existing frameworks to fit the Chinese organizational context. Talent ecosystem characteristics were measured using a 12-item scale, capturing three dimensions: talent density (4 items), talent flow (4 items), and talent structure (4 items). Sample items include ‘Our organization has a high concentration of talented individuals in key positions’ (talent density), ‘There is active movement of talent across different units in our organization’ (talent flow), and ‘Our workforce encompasses diverse skills and expertise’ (talent structure). Responses were recorded on a 5-point Likert scale ranging from 1 (strongly disagree) to 5 (strongly agree).

Green innovation capability was measured using a 9-item scale, capturing three dimensions: green product innovation (3 items), green process innovation (3 items), and green management innovation (3 items). Organizational learning was measured using an 8-item scale, capturing both exploitative learning (4 items) and exploratory learning (4 items). Scientific leadership was measured using a 6-item scale developed based on Liang (2024), capturing the key dimensions of scientific leadership including technical expertise, systems thinking, and talent development orientation. This scale was adapted from Liang's (2024) research, which developed and validated the measure through extensive qualitative research in Chinese organizations. The scale captures three theoretically-derived dimensions: (1) evidence-based decision-making and methodological rigor, (2) systems thinking and integrative capability, and (3) talent development orientation. Sample items include: ‘Our leaders make decisions based on systematic data analysis rather than intuition alone’ (evidence-based); ‘Our leaders can integrate insights from multiple technical domains to address complex problems’ (systems thinking); and ‘Our leaders actively mentor emerging talent and create opportunities for next-generation development’ (talent development).

### 4.3 Analytical approach

Data analysis was conducted using structural equation modeling (SEM) with SmartPLS 4.0 software. PLS-SEM was chosen for its advantages in handling complex models with multiple constructs and mediating/moderating effects, as well as its less stringent requirements regarding sample size and distributional assumptions (Hair et al., 2019). The analysis followed a two-step approach: first examining the measurement model to establish reliability and validity, then testing the structural model to evaluate hypotheses. This two-step approach is consistent with best practices in SEM research and ensures that the measurement instruments are sound before testing the theoretical relationships.

To test the mediating effect of organizational learning (H2), it employed the bootstrapping procedure with 5,000 resamples to generate confidence intervals for the indirect effects. Bootstrapping is preferred over the traditional Baron and Kenny approach because it does not assume normality of the sampling distribution and provides more accurate estimates of indirect effects. The moderating effect of scientific leadership (H3) was tested using the product indicator approach, with simple slope analysis conducted to interpret significant interaction effects. The product indicator approach is appropriate for PLS-SEM and allows for the examination of how the relationship between independent and dependent variables changes at different levels of the moderator.

Control variables included firm size (measured as the natural logarithm of employee count), firm age (measured in years since establishment), and industry sector (dummy variables for biotechnology, new energy, and advanced manufacturing, with information technology as the reference category). These controls were included to account for potential confounding effects that might influence the relationships of interest.

## 5 Results and discussion

### 5.1 Measurement model assessment

The measurement model was assessed using standard criteria for reliability and validity. Composite reliability (CR) values for all constructs exceeded the recommended threshold of 0.70, ranging from 0.82 to 0.91, indicating satisfactory internal consistency. Average variance extracted (AVE) values ranged from 0.58 to 0.72, all exceeding the 0.50 threshold, demonstrating adequate convergent validity. These results suggest that the measurement items reliably capture the intended constructs and that the constructs explain a substantial proportion of variance in their indicators.

Discriminant validity was assessed using the Fornell-Larcker criterion and the heterotrait-monotrait (HTMT) ratio. The square root of AVE for each construct exceeded its correlations with other constructs, and all HTMT ratios were below the conservative threshold of 0.85, supporting discriminant validity (Henseler et al., 2015). The results indicate that the constructs are distinct from one another and that the measurement model adequately distinguishes between the different theoretical concepts. Factor loadings for all items exceeded 0.70, further supporting the reliability and validity of the measurement instruments. Table 1 shows descriptive statistics and correlation matrix.

**Table 1:** Descriptive statistics and correlation matrix.

Variable	Mean	SD	1	2	3	4	5	6	7
1. Talent density	3.72	0.84							
2. Talent flow	3.58	0.91	0.52***						
3. Talent structure	3.65	0.88	0.48***	0.55***					
4. Organizational learning	3.81	0.79	0.45***	0.42***	0.51***				
5. Scientific leadership	3.45	0.95	0.38***	0.35***	0.41***	0.58***			
6. Green innovation capability	3.62	0.87	0.47***	0.44***	0.49***	0.62***	0.53***		
7. Firm size	3.15	1.02	0.12	0.08	0.15*	0.11	0.09	0.14*	
8. Firm age	2.89	1.15	0.09	0.06	0.11	0.08	0.07	0.10	0.22**

*N* = 287. *p < 0.05, * *p < 0.01, * **p < 0.001. Diagonal elements (in parentheses, not shown in this table) are the square roots of AVE. Firm size measured as natural logarithm of employee count. Firm age measured in years since establishment.

### 5.2 Structural model and hypothesis testing

The structural model demonstrated satisfactory fit indices. The standardized root mean square residual (SRMR) was 0.048, below the 0.08 threshold, indicating good model fit. The coefficient of determination (R^2^) for green innovation capability was 0.62, indicating that the model explains a substantial proportion of variance in the dependent variable. The R^2^ values for organizational learning and talent ecosystem characteristics were 0.54 and 0.38, respectively, suggesting that the model adequately captures the antecedents of these key constructs.

Hypothesis 1 proposed a positive relationship between talent ecosystem characteristics and green innovation capability. The results supported this hypothesis (*β* = 0.47, *p* < 0.001), indicating that firms with stronger talent ecosystems exhibit higher levels of green innovation capability. This finding aligns with the theoretical expectation that human capital resources provide the foundation for sustainable innovation. The effect size (f^2^ = 0.28) indicates a medium to large effect, suggesting that talent ecosystem characteristics have a practically significant impact on green innovation capability.

Hypothesis 2 posited that organizational learning mediates the relationship between talent ecosystem and green innovation. The indirect effect was significant (*β* = 0.21, 95% CI [0.13, 0.31]), supporting the mediating role of organizational learning. The confidence interval does not include zero, indicating that the mediation effect is statistically significant at the 0.05 level. This finding suggests that talent ecosystem characteristics enhance green innovation capability partly by facilitating organizational learning processes. The proportion of the total effect mediated by organizational learning was approximately 31%, indicating partial mediation.

**Figure 2:**
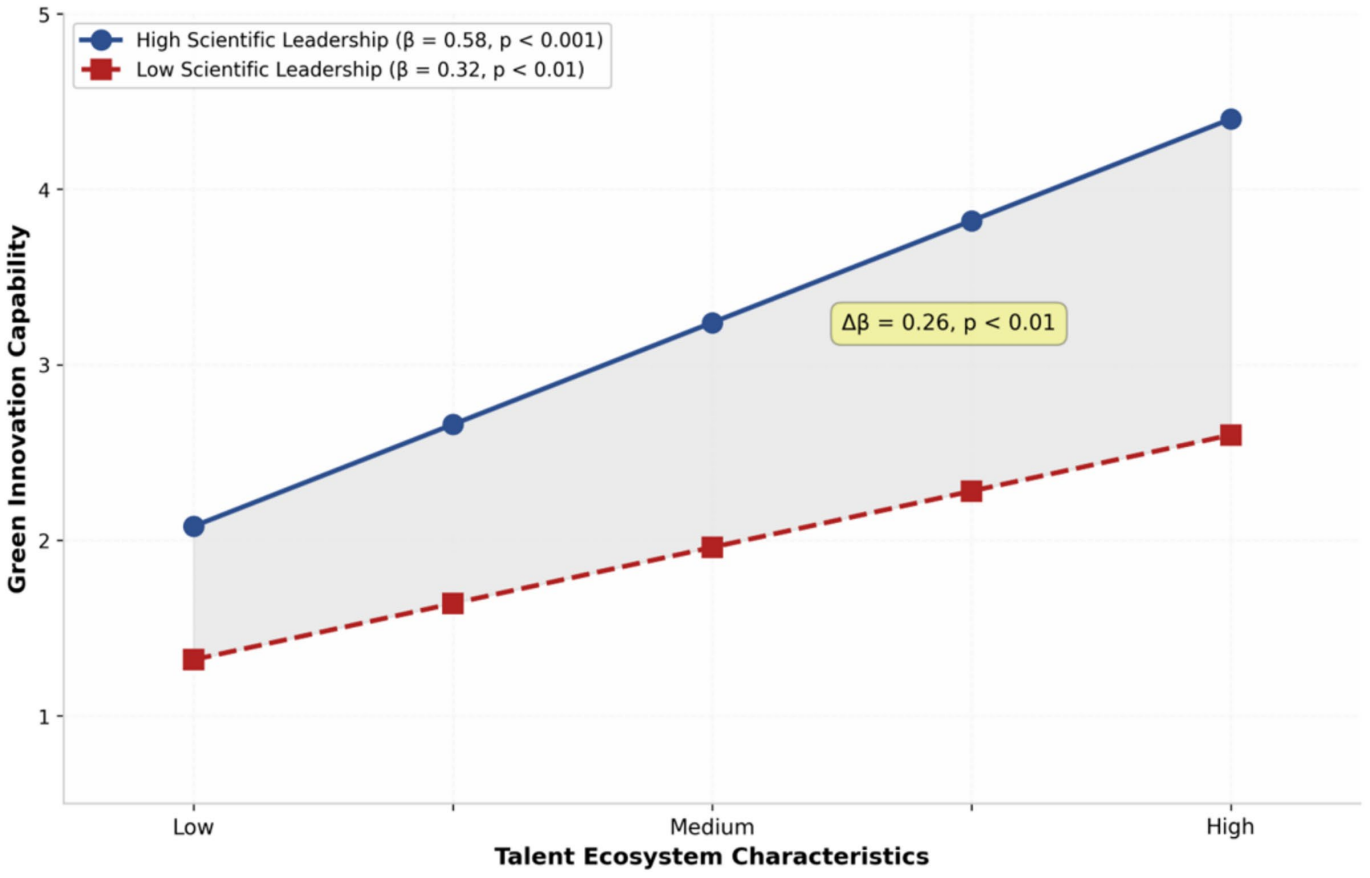
The moderating effect of scientific leadership on the talent ecosystem-green innovation relationship.

Hypothesis 3 proposed that scientific leadership moderates the talent ecosystem-green innovation relationship. The interaction term was significant (*β* = 0.18, *p* < 0.01), supporting the moderating effect. Simple slope analysis revealed that the relationship between talent ecosystem and green innovation was stronger at high levels of scientific leadership (*β* = 0.58, *p* < 0.001) compared to low levels (*β* = 0.32, *p* < 0.01). The difference between these slopes was statistically significant (Δβ = 0.26, *p* < 0.01), confirming that scientific leadership amplifies the positive relationship between talent ecosystem characteristics and green innovation capability (as shown in Figure 2).

### 5.3 Discussion

The findings of this study contribute to our understanding of the mechanisms through which talent ecosystems influence sustainable innovation outcomes. The significant direct effect of talent ecosystem characteristics on green innovation capability supports the theoretical proposition that human capital serves as a fundamental resource for environmental sustainability. This finding extends the resource-based view by demonstrating how the configuration and dynamics of human resources, rather than merely their accumulation, create innovation capabilities. The results suggest that organizations seeking to enhance their green innovation performance should focus not only on attracting talented individuals but also on creating the conditions that enable talent to flourish and interact productively.

The direct path (H1) from talent ecosystem characteristics to green innovation capability may suffer from omitted variable bias. Green innovation often correlates strongly with regulatory pressure, industry dynamism, and slack resources—factors that simultaneously attract talent. The model risks conflating talent effects with institutional isomorphism, where firms in stringent regulatory environments both invest in green innovation and compete for the same talent pool. Reverse causality also merits consideration: firms with strong green innovation reputations may attract higher-quality talent, creating a virtuous cycle that the cross-sectional design cannot disentangle.

The mediation through organizational learning (H2) assumes a linear, absorptive capacity logic. However, these learning modes may be substitutes rather than complements in resource-constrained settings. High talent density could paradoxically reduce organizational learning effectiveness if it increases coordination costs or fosters knowledge hoarding rather than sharing. The model does not specify whether exploitative and exploratory learning operate sequentially or simultaneously — a critical distinction given their inherent tensions.

The scientific leadership moderation (H3) is under-theorized. Knowledge integration as a moderator implies that scientific leadership amplifies the talent-learning relationship, yet alternative configurations exist. Under conditions of high environmental uncertainty, scientific leadership might buffer organizations from the need for broad talent ecosystems by providing decisive technological direction. Conversely, in mature industries with established green standards, scientific leadership may become redundant if best practices are already codified.

## 6 Conclusion

### 6.1 Theoretical implications

This study makes several contributions to the literature on sustainability management and strategic human resource management. First, it extends talent ecosystem theory by demonstrating its applicability to the domain of environmental sustainability. While previous research has focused primarily on the relationship between talent ecosystems and economic performance, this study shows that ecosystem characteristics also influence green innovation capabilities, suggesting broader implications for organizational sustainability (Alshukri et al., 2024). By connecting talent ecosystem theory with the green innovation literature, this research opens new avenues for understanding how human capital development can contribute to environmental goals. By emphasizing talent density, flow, and structural diversity as antecedents of innovation, this study advances understanding of workplace creativity as an emergent property of interconnected talent networks rather than merely individual attributes. This study answers calls to examine how structural features of talent systems shape creative capacity through psychological mechanisms such as intrinsic motivation, complementary fit, and creative self-efficacy (Zhao and Ren, 2025; Qian et al., 2025).

Second, the study integrates insights from leadership research and innovation management to develop a more nuanced understanding of how human capital translates into sustainable innovation outcomes. The identification of organizational learning as a mediating mechanism and scientific leadership as a moderating factor provides a more complete picture of the micro-foundations of green innovation capability. Specifically, organizational learning represents the psychological and team-level mechanism through which talent ecosystem characteristics translate into innovation outcomes, while scientific leadership creates the psychological safety and intellectual stimulation that enable teams to leverage their collective capabilities effectively. This integrative approach responds to calls for research that bridges multiple levels of analysis and examines the processes through which organizational resources are converted into innovation outcomes (Lawson and Samson, 2001). This study indicates that knowledge sharing and creative self-efficacy serve as critical team-level mechanisms linking leadership to innovative behavior (Ni et al., 2025), and that team resilience capabilities—encompassing positive consensus and adaptability to change—constitute foundational attributes of future-ready teams (Hsu et al., 2025).

Third, the study contributes to the growing body of research on sustainability in emerging economies by providing empirical evidence from the Chinese context. The findings suggest that the mechanisms linking talent management to green innovation may have some universal applicability while also being shaped by specific institutional and cultural factors. This contextual sensitivity is important for developing theories that are both generalizable and sensitive to local conditions.

### 6.2 Practical implications

The findings of this study offer several practical implications for managers seeking to enhance their organizations’ green innovation capabilities. First, investments in talent ecosystem development should be viewed as strategic priorities for sustainability. This includes not only recruiting talented individuals but also creating conditions that enable talent density, flow, and structural diversity to generate innovation outcomes. Managers should assess their current talent ecosystems and identify areas for improvement, such as increasing the concentration of talented individuals in key positions, facilitating internal mobility and knowledge transfer, and building more diverse teams with complementary expertise. These practices contribute to building future-ready teams capable of adaptive and innovative work by ensuring continuous knowledge refreshment, cognitive diversity, and collaborative capacity. Such configurations cultivate team resilience and adaptive capability, reflecting the core attributes of future-ready teams that thrive under dynamic and uncertain conditions (Hsu et al., 2025).

Second, organizations should invest in building organizational learning capabilities as a complement to talent development initiatives. This includes creating knowledge management systems, fostering collaborative cultures, and establishing processes that facilitate the sharing and integration of diverse expertise relevant to environmental sustainability. Learning should be viewed not as an individual activity but as an organizational process that requires deliberate design and management. Organizations can benefit from creating communities of practice, establishing cross-functional teams, and implementing after-action reviews that capture and disseminate lessons learned from green innovation projects.

Third, the development of scientific leadership should be prioritized, particularly in knowledge-intensive contexts where green innovation is critical. Organizations should identify and cultivate leaders with deep technical expertise, systems thinking capability, and a commitment to developing the next generation of talent. Leadership development programs should emphasize not only administrative and interpersonal skills but also technical depth and the ability to integrate diverse perspectives. Organizations can benefit from creating career paths that allow technical experts to advance into leadership positions without abandoning their technical identities.

### 6.3 Limitations and future research

This study has several limitations that suggest directions for future research. First, the cross-sectional design limits our ability to draw causal inferences about the relationships examined. While the theoretical framework proposes causal relationships, the cross-sectional data cannot definitively establish causality. Longitudinal studies would provide stronger evidence regarding the causal mechanisms linking talent ecosystems to green innovation outcomes and would allow researchers to examine how these relationships evolve over time.

Second, the study focused on knowledge-intensive firms in China, which may limit the generalizability of findings to other contexts. While the Chinese context offers valuable insights, the specific institutional and cultural factors at play may differ in other countries and regions. Future research should examine whether the proposed relationships hold in different institutional settings, industry sectors, and cultural contexts. Comparative studies across countries could provide valuable insights into how national contexts shape the relationship between talent management and green innovation.

Third, while this study examined organizational learning as a mediator and scientific leadership as a moderator, other mechanisms and boundary conditions may also be relevant. Future research could explore additional mediating processes such as organizational culture, knowledge networks, and innovation climate, as well as other moderating factors such as environmental dynamism and resource availability. The integration of additional variables could provide a more comprehensive understanding of the complex relationships among talent ecosystems, leadership, and green innovation.

In conclusion, this study demonstrates that talent ecosystems serve as critical foundations for green innovation capability in knowledge-intensive firms. By developing robust talent ecosystems, fostering organizational learning, and cultivating scientific leadership, organizations can enhance their capacity for sustainable innovation, contributing to both competitive advantage and environmental sustainability. As firms around the world grapple with the challenges of sustainable development, the insights from this research provide guidance for leveraging human capital as a driver of green transformation.

## Data Availability

The original contributions presented in the study are included in the article/supplementary material, further inquiries can be directed to the corresponding author.
